# Complete Anterior Tibial Tendon Rupture After Closed Tibial Shaft Fracture: A Case Report

**DOI:** 10.1155/cro/3083462

**Published:** 2026-08-23

**Authors:** Juanita Villalba Reyes, Juanita Fetecua Chaparro, Julián Salavarrieta Varela, Diana Paola Montoya Murillo, Nicolás Jiménez Arrieta

**Affiliations:** ^1^ Orthogeriatrics and Trauma Service, Santa Fe de Bogotá Foundation University Hospital, Bogotá, Colombia; ^2^ Foot and Ankle Service, Santa Fe de Bogotá Foundation University Hospital, Bogotá, Colombia; ^3^ Santa Fe de Bogotá Foundation University Hospital, Bogotá, Colombia

**Keywords:** anterior tibial tendon, care guidelines, rehabilitation, surgical repair, tendon rupture, tibial fracture

## Abstract

Acute anterior tibialis tendon (ATT) rupture is a rare injury that may go unrecognized in the acute trauma setting, particularly when it co‐occurs with a closed tibial shaft fracture. We report a case of complete ATT rupture in a 25‐year‐old male following a motorcycle accident causing a distal tibial shaft fracture. The rupture was identified intraoperatively; during distal locking screw placement, tendon‐like fibers were observed through the surgical incision. Given this unexpected finding, the incision was extended to allow further exploration. Careful dissection revealed a complete rupture of the anterior tibial tendon, which had not been identified preoperatively. Because of significant soft‐tissue edema at the time of fracture fixation, a staged surgical approach was adopted: temporary proximal stump fixation to the superior extensor retinaculum followed by definitive end‐to‐end repair 10 days later using Krakow and modified Kessler sutures. At 6 months, MRI confirmed tendon continuity, dorsiflexion strength was 4/5, and the AOFAS score was 90 (excellent). This case illustrates the diagnostic challenge of ATT rupture concurrent with tibial fracture and highlights that opportunistic visualization of the tendon may occur during distal locking procedures when the fracture apex is anteriorly oriented—an anatomical coincidence that, when recognized, enables timely repair. The principal clinical lessons are as follows: (1) the importance of including ATT integrity in the assessment of tibial fractures with anterior apex angulation or dorsiflexion deficit; (2) a staged approach is feasible and effective when soft‐tissue conditions preclude immediate repair; and (3) early surgical repair, when stumps are viable, is associated with excellent functional outcomes.

## 1. Introduction

Acute ruptures of the anterior tibial tendon are uncommon injuries, first described by Brüning in 1905 [1]. They are generally associated with minor trauma in patients over 45 years of age with predisposing comorbidities such as diabetes, hypothyroidism, or gout [2]. The ATT accounts for up to 80% of ankle dorsiflexion; its loss results in steppage gait and compensatory overload of the extensor hallucis longus and extensor digitorum longus muscles [1].

When the injury occurs in the context of a closed tibial shaft fracture, it represents a particularly challenging diagnostic scenario. The mechanism of direct laceration by a displaced bone fragment at the fracture apex may go unnoticed during initial evaluation: pain, swelling, and limited range of motion obscure the clinical signs of dorsiflexion deficit, and a tendon injury is seldom suspected when attention is focused on the bony injury [3–5]. The limited existing literature describes fewer than 10 cases of this specific injury combination, making it a truly rare clinical entity.

Recent literature emphasizes that early diagnosis and treatment are crucial to prevent persistent functional deficit. MRI and dynamic ultrasound have demonstrated high sensitivity for identifying tendon discontinuity [2, 6]. In this report, we discuss the clinical reasoning process, the decision‐making around surgical timing, and the functional outcomes achieved, aiming to contribute to the body of evidence that can guide surgeons facing this uncommon diagnostic and therapeutic challenge.

## 2. Case Presentation

A 25‐year‐old male with no significant medical history presented following a motorcycle traffic accident. He reported severe right leg and ankle pain, swelling, and deformity, with the inability to bear weight. On admission, he was hemodynamically stable; pain intensity was 6/10. Physical examination revealed deformity at the distal third of the right leg and ankle, bimalleolar edema, ecchymosis, and limited range of motion due to pain, without neurovascular deficit. The possibility of a dorsiflexion deficit was clinically suspected given the anterior apex deformity noted on examination, though it could not be formally assessed given the level of pain and swelling.

**Figure 1:**
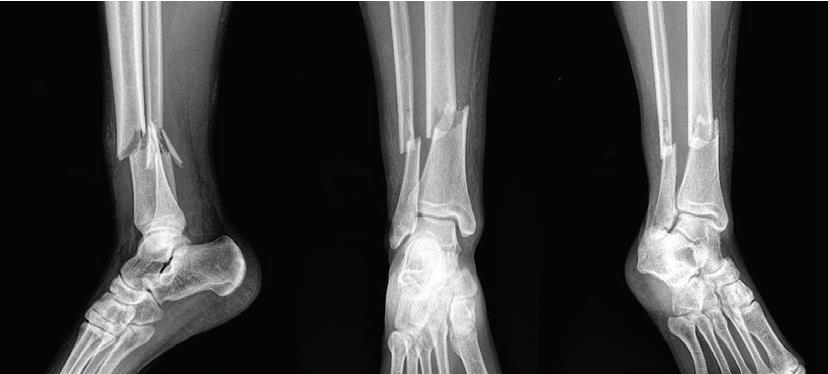
Right ankle radiograph (AP, lateral, and internal rotation views) showing a short oblique fracture of the distal third of the tibial shaft with valgus and procurvatum angulation, associated with a comminuted distal fibular fracture.

**Figure 2:**
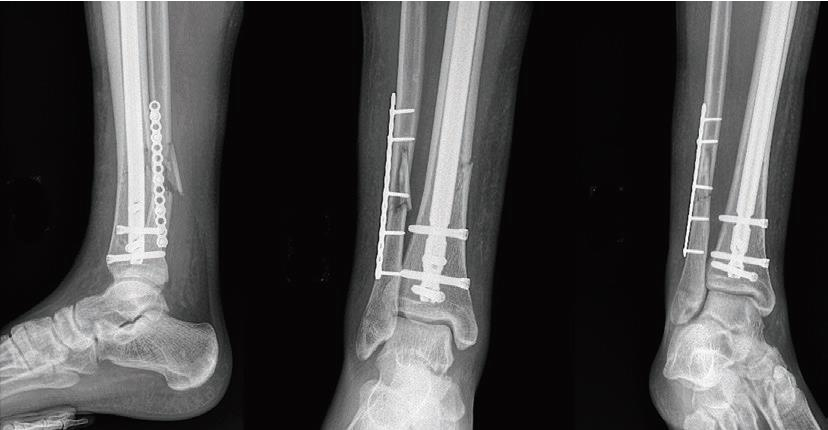
Postoperative radiographs showing intramedullary tibial nailing and fibular plate fixation.

Radiographs confirmed a short oblique distal‐third tibial shaft fracture with valgus and procurvatum angulation associated with a comminuted distal fibular fracture (Figure 1). Initial management included immobilization and analgesics. Definitive osseous fixation was performed 48 h after admission using an intramedullary tibial nail and fibular plate fixation (Figure 2).

During placement of the distal locking screws, the operative incision was made over the distal anterior tibia, in proximity to the fracture apex. At this site, a complete ATT rupture was directly visualized. The tendon was disrupted at the level of the procurvatum deformity, consistent with a laceration mechanism caused by the displaced anterior apex of the fracture. It should be noted that this visualization was not the result of a planned systematic exploration of the tendon, but rather an opportunistic finding resulting from the anatomical coincidence of the distal locking incision location and one of the stumps of the ruptured tendon, a circumstance specific to this fracture pattern that should not be extrapolated as a general recommendation for routine tendon inspection in all tibial shaft nailing procedures.

Given the significant soft‐tissue edema present at the time of fracture fixation, immediate end‐to‐end tendon repair was considered technically inadvisable due to the risks of repair failure and wound complications. A staged approach was therefore selected: the proximal stump was temporarily fixed to the superior extensor retinaculum to prevent retraction, and the wound was closed with incisional negative pressure wound therapy (iNPWT), with definitive repair planned after edema resolution.

**Figure 3:**
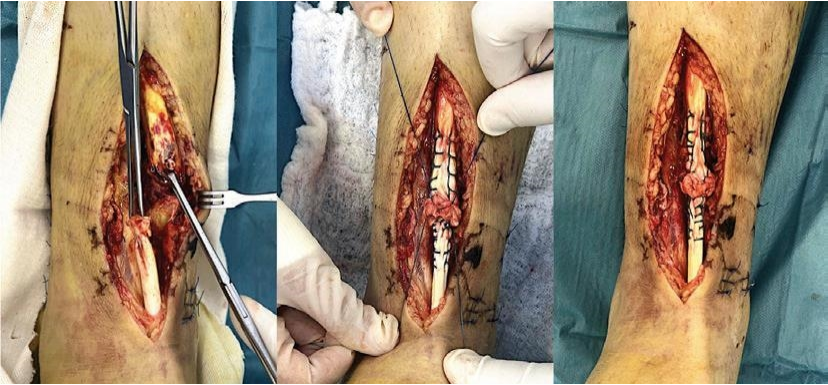
Intraoperative photographs showing complete anterior tibial tendon rupture and end‐to‐end reconstruction with Krakow and modified Kessler sutures.

Ten days later, once the soft‐tissue envelope had recovered, definitive end‐to‐end repair was performed using Krakow and modified Kessler sutures, achieving adequate stump apposition (Figure 3). Postoperative immobilization was maintained in neutral dorsiflexion.

**Figure 4:**
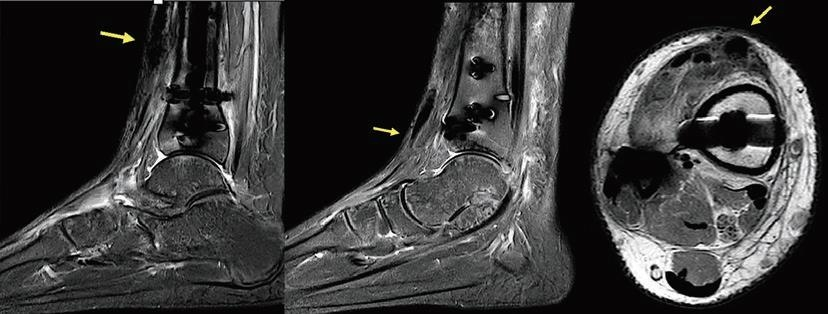
MRI demonstrating postoperative continuity of the anterior tibial tendon.

Postoperative recovery was uneventful. At the 2‐week check, wounds were properly healed. A long‐leg nonarticulated walker boot was used for 6 weeks with progressive weight‐bearing. Formal ankle mobilization was initiated at Week 8. MRI at 3 and 6 months confirmed bone consolidation and tendon continuity (Figure 4). At 6 months, dorsiflexion strength was 4/5 and the AOFAS score was 90, reflecting excellent functional recovery. A structured patient timeline is presented in Table 1.

**Table 1:** Structured patient timeline.

Timepoint	Events/findings	Interventions
Admission (Day 0)	25 yo male, MVC motorcycle, right leg/ankle pain, edema, deformity, neurovascularly intact, pain 6/10 XR: distal 1/3 tibial shaft fracture + comminuted fibular fracture	Immobilization, analgesics, initial assessment
Day 2 (48 h post‐admission)	Hemodynamic stability maintained; No compartment syndrome; Soft‐tissue edema present	Intramedullary tibial nailing + fibular plate fixation; Intraoperative findings: complete ATT rupture at fracture apex, temporary stump fixation to superior extensor retinaculum, iNPWT wound closure
Day 12 (10 days post‐nailing)	Soft‐tissue edema resolved; Wounds intact	Definitive end‐to‐end ATT repair (Krakow + modified Kessler sutures); Immobilization in neutral dorsiflexion
Week 2 (post‐repair)	Wound intact	Long‐leg nonarticulated walker boot, partial weight‐bearing initiated
Week 8	Progressive clinical improvement; No wound complications	Ankle mobilization started; Dorsiflexion re‐education exercises
3 months	MRI: bone consolidation + tendon continuity confirmed	Strengthening protocol continued
6 months	Dorsiflexion strength 4/5, AOFAS score 90 (excellent)	Functional reintegration; Discharge from structured follow‐up

Abbreviations: AOFAS: American Orthopaedic Foot and Ankle Society score; ATT: anterior tibial tendon; iNPWT: incisional negative pressure wound therapy; MVC: motor vehicle collision.

## 3. Discussion

ATT rupture concurrent with closed tibial shaft fracture is an exceptionally rare injury. Fewer than 10 cases have been described in the indexed literature; in most of them, as in ours, the correct diagnosis was made only intraoperatively or after a period of diagnostic delay [3–5]. The rarity itself constitutes a diagnostic challenge: surgeons are not primed to suspect tendon injury in the absence of an open wound.

The key diagnostic pitfall in this scenario is that ATT rupture may be clinically occult. The swelling, pain, and deformity associated with the fracture mask the signs that would otherwise prompt suspicion: inability to actively dorsiflex the ankle and the absence of a palpable tendon under the skin. Moreover, because plantarflexion and inversion strength are preserved via intact posterior tibial tendon function, the patient may not volunteer a complaint attributable to anterior compartment dysfunction [1, 3]. For this reason, in tibial shaft fractures with anterior apex angulation or procurvatum deformity, active assessment of dorsiflexion should be attempted whenever pain permits, and any deficit should prompt pre‐ or intraoperative imaging. Once the anesthetic block of the affected limb has been performed, an intraoperative ultrasound examination could be conducted. MRI and dynamic ultrasound have demonstrated high sensitivity for detecting tendon discontinuity and should be requested when clinical suspicion arises [2, 6].

Regarding intraoperative decision‐making: in our case, the ATT rupture was identified fortuitously during the distal locking incision, which coincided anatomically with the localization of one of the stumps of the tibialis anterior tendon (this occurs because the interlocking of the nail is distal to the fracture site). This situation, where the distal locking approach directly overlies the injury site, may occur specifically when fractures present with anteriorly directed apex deformity at the distal third of the tibial shaft. We do not advocate for routine deliberate exploration of the ATT during all tibial shaft nailing procedures; the standard minimally invasive incisions for distal locking would not allow adequate visualization in most cases. However, when the fracture morphology (anterior procurvatum apex) raise suspicion preoperatively, preoperative MRI or intraoperative direct exploration through the locking incision, applying traction to the tibialis anterior tendon, if visible, may be considered to achieve timely identification and repair.

Once the diagnosis was confirmed, the principal therapeutic decision was surgical timing. Immediate tendon repair was judged inadvisable given the degree of soft‐tissue edema, which would have increased the risk of wound dehiscence and repair failure. A staged approach, temporary stump fixation to prevent retraction, followed by definitive repair after edema resolution, proved effective. The patient’s eventual AOFAS score of 90 is consistent with the best functional outcomes reported in the literature for early end‐to‐end repair (90–95 points; 12–16 weeks return to activity), supporting the value of timely surgical management even when an intermediary stage is required [1, 2, 7].

Regarding functional outcomes in distal tibial trauma more broadly, Guzel et al. [8] reported that early surgical fixation in pilon fractures was associated with better AOFAS scores and shorter healing times, reinforcing the principle that timely intervention in distal tibial injuries, whether osseous or tendinous, is associated with improved recovery. Although their study addresses a different injury type, the functional outcome framework is directly relevant to the decision‐making context of our case.

An untreated or delayed ATT rupture carries important long‐term sequelae: steppage gait, chronic anterior ankle pain, flatfoot deformity, difficulty navigating uneven terrain, and increased fall risk [1, 3, 5]. Chronic tendon insufficiency also compromises the effectiveness of delayed reconstruction, which is associated with longer rehabilitation and less complete functional recovery compared with primary repair [9]. In chronic or complex cases with large defects, tendon transfers (EHL or EDL) or autografts (semitendinosus) remain appropriate alternatives [2, 7, 9]. Surgical technique options and functional outcomes from the literature are summarized in Tables 2 and 3, respectively.

**Table 2:** Surgical techniques for complete ATT rupture.

Technique	Indications	Advantages	Limitations	Sources
End‐to‐end repair	Acute ruptures with viable stumps	Restores length and physiological vector	Not applicable in large defects	[1, 2]
Reinsertion with bone anchors	Distal avulsion	Good fixation, restores tension	Requires adequate bone	[1, 7]
Split or turn‐down flap of the TTA	Moderate or chronic defect	Avoids donor site, good integration	Reduced final strength	[2, 7]
Semitendinosus autograft	Large or chronic defects	Sufficient length, biocompatible	Donor site morbidity	[2, 9]
Intercalated allograft	Extensive defects	No donor site morbidity	Longer integration time, cost	[1, 7, 9]
EHL transfer	Irreparable injuries	Simple technique	Reduced strength and control	[7]

*Note:* Sources: Chen et al. 2021 [1], Tickner et al. 2018 [7], Givissis et al. 2004 [3], Ebrahimi et al. 2010 [4], Lefebvre et al. 2011 [6], Christman‐Skieller et al. 2015 [2], and Otte et al. 2002 [9].

**Table 3:** Functional outcomes by surgical technique.

Technique	Scales used	Average outcome	Time to return to activity	Sources
End‐to‐end repair	AOFAS, FAOS	90–95 points	12–16 weeks	[1, 2, 7]
Split TTA flap	AOFAS	85–90 points	16–20 weeks	[2, 7]
Semitendinosus autograft	AOFAS, FAOS	88–92 points	18–24 weeks	[2, 9]
EHL transfer	AOFAS	75–85 points	>24 weeks	[2, 7]

*Note:* Sources: Chen et al. 2021 [1], Tickner et al. 2018 [7], Christman‐Skieller et al. 2015 [2], and Otte et al. 2002 [9].

The principal limitations of this report are those inherent to any single case: the absence of a control group, potential for center‐specific surgical factors to influence outcomes, and a follow‐up limited to 6 months. Additionally, the staged repair, while effective here, introduced a period during which tendon retraction was a risk; our management of this with retinacular fixation and iNPWT provides a potential template for others but requires prospective validation. Further studies with larger series and longer follow‐up are needed to establish standardized recommendations for this injury combination.

## 4. Conclusions

This case reaffirms that ATT rupture can occur as a concealed injury in closed tibial shaft fractures, particularly those with anterior apex angulation or procurvatum deformity. Clinical suspicion, based on fracture morphology and a careful assessment of dorsiflexion function, if tolerated by the patient, is the critical first step in timely diagnosis. Furthermore, in cases of distal tibial shaft fractures, it is advisable to routinely evaluate active ankle dorsiflexion following fracture fixation, once the effects of the anesthetic motor block on the limb have resolved. When the distal locking approach coincides anatomically with the course of the tibialis anterior tendon, opportunistic visualization and manipulation with traction to confirm its integrity may reveal the injury; this finding, while not a basis for recommending routine tendon exploration in all tibial nailing procedures, underscores the value of heightened vigilance in susceptible fracture patterns. A staged surgical approach is a viable option when immediate repair is contraindicated by soft‐tissue status. Early repair, even when performed in a delayed‐staged fashion, is associated with excellent functional outcomes, consistent with the broader literature on distal tibial trauma management.

Surgeons should maintain a high index of suspicion when fractures characteristics, soft‐tissue findings, or postoperative functional deficits suggest possible tendon injury. Direct tendon inspection may be considered only when clinical findings raise concern, rather than as a routine component of intramedullary nailing.

Although previous reports have described anterior tibial tendon rupture associated with tibial shaft fractures, the injury remains exceedingly rare and frequently overlooked [3–5, 9, 10]. This report contributes additional evidence regarding the diagnosis challenges, imaging findings, surgical management, and postoperative functional recovery in this uncommon injury pattern.

## Funding

No funding was received for this manuscript.

## Disclosure

All medical information and case details were written and verified entirely by the authors.

## Ethics Statement

This case report was prepared in accordance with the CARE (CAse REport) guidelines for reporting case reports. A complete CARE checklist is provided in Table S1.

## Consent

Written informed consent for publication was obtained from the patient.

## Conflicts of Interest

The authors declare no conflicts of interest.

## Supporting information


**Supporting Information** Additional supporting information can be found online in the Supporting Information section. Table S1: The complete CARE (CAse REport) guideline checklist, mapping each reporting item to the corresponding section or page of this manuscript.

## Data Availability

Data sharing not applicable—this article describes a single clinical case and no datasets were generated or analyzed.

## References

[1] Chen J. , Kadakia R. , Akoh C. C. , and Schweitzer K. M. , Management of Anterior Tibialis Tendon Ruptures, Journal of the American Academy of Orthopaedic Surgeons. (2021) 29, no. 16, 691–701, 10.5435/JAAOS-D-20-00802. 34197343

[2] Christman-Skieller C. , Merz M. K. , and Tansey J. P. , A Systematic Review of Tibialis Anterior Tendon Rupture Treatments and Outcomes, American Journal of Orthopedics. (2015) 44, no. 3, E49–E54, (no DOI has been assigned to this article). 25844597

[3] Givissis P. , Christodoulou A. , Karataglis D. , Terzidis I. , and Pournaras J. , Laceration of Tibialis Anterior Tendon Complicating a Closed Tibial Fracture: A Case Report, Journal of Foot and Ankle Surgery. (2004) 43, no. 6, 426–429, 10.1053/j.jfas.2004.09.010, 15605058. 15605058

[4] Ebrahimi F. V. , Tofighi M. , and Khatibi H. , Closed Tibial Fracture Associated With Laceration of Tibialis Anterior Tendon, Journal of Foot and Ankle Surgery. (2010) 49, no. 1, 86.e19–86.e22, 10.1053/j.jfas.2009.08.013, 19906544. 19906544

[5] Rad S. B. , Sadighi M. , Kafiabadi M. J. , Ebrahimpour A. , Pourmahmoudian M. , and Karami A. , Tibialis anterior and extensor hallucis longus tendon ruptures complicating a closed tibial fracture: A case report, International Journal of Surgery Case Reports. (2024) 124, no. C, 110388, 10.1016/j.ijscr.2024.110388, 39357482. 39357482 PMC11471250

[6] Lefebvre B. , Beldame J. , Bertiaux S. , and Biga N. , Open and Subcutaneous Recent Tibialis Anterior Tendon Ruptures: Does Postoperative Immobilization Method Influence Outcome?, Orthopaedics & Traumatology: Surgery &amp; Research. (2011) 97, no. 2, 211–216, 10.1016/j.otsr.2010.09.018, 21273155. 21273155

[7] Tickner A. , Thorng S. , Martin M. , and Marmolejo V. , Management of Isolated Anterior Tibial Tendon Rupture: A Systematic Review and Meta-Analysis, Journal of Foot and Ankle Surgery. (2019) 58, no. 2, 213–220, 10.1053/j.jfas.2018.08.001, 30554867. 30554867

[8] Güzel I. , Ulusoy I. , Yilmaz M. , Tantekin M. F. , and Kivrak A. , Early Versus Delayed Plate Fixation in Pilon Fractures—Which Is More Advantageous?, Journal of the American Podiatric Medical Association. (2026) 116, no. 2, 25087, 10.7547/25-087, 42042524. 42042524

[9] Otte S. , Klinger H. M. , Lorenz F. , and Haerer T. , Operative Treatment in Case of a Closed Rupture of the Anterior Tibial Tendon, Archives of Orthopaedic and Trauma Surgery. (2002) 122, no. 3, 188–190, 10.1007/s004020100346, 11928008. 11928008

[10] Anagnostakos K. , Bachelier F. , Fürst O. A. , and Kelm J. , Rupture of the Anterior Tibial Tendon: Three Clinical Cases, Anatomical Study, and Literature Review, Foot & Ankle International. (2006) 27, no. 5, 330–339, 10.1177/107110070602700504, 16701053. 16701053

