# Supplementary material for: Complete Anterior Tibial Tendon Rupture After Closed Tibial Shaft Fracture: A Case Report
Source: Case Rep Orthop. 2026 Aug 23;2026:3083462. doi: 10.1155/cro/3083462 (PMC13500996; doi:10.1155/cro/3083462)
Supplement: Supplementary file 1 [file CRO-2026-3083462-s001.docx]

**Supplementary Table S1. CARE Guideline Checklist**

| **CARE Item** | **Section/Page** |
| --- | --- |
| 1. Title – contains the words 'case report' | Title |
| 2. Key Words – 2 to 5 key words | Abstract |
| 3a. Abstract: Introduction – what is unique about this case? | Abstract |
| 3b. Abstract: The patient's main concern and clinical findings | Abstract |
| 3c. Abstract: Diagnoses, interventions, and outcomes | Abstract |
| 3d. Abstract: Conclusion – what are the main lessons learned? | Abstract |
| 4. Introduction – adequate background and context for this case | Introduction |
| 5a. Patient information – de-identified demographic/relevant info | Case Presentation |
| 5b. Chief complaint and reason for seeking care | Case Presentation |
| 5c. Medical, family, and psychosocial history | Case Presentation |
| 5d. Relevant past interventions and their outcomes | Case Presentation |
| 6a. Clinical findings – physical examination | Case Presentation |
| 6b. Key clinical findings | Case Presentation |
| 7. Timeline – depicted in structured table | Patient Timeline (Table 1) |
| 8a. Diagnostic assessment – methods and results | Case Presentation |
| 8b. Diagnostic reasoning including differential diagnosis | Case Presentation / Discussion |
| 8c. Prognostic characteristics when applicable | Discussion |
| 9a. Therapeutic intervention – type of intervention | Case Presentation |
| 9b. Administration and intensity of intervention | Case Presentation |
| 9c. Changes in interventions with justification | Case Presentation |
| 10a. Follow-up and outcomes – clinician-assessed outcomes | Case Presentation |
| 10b. Patient-reported outcomes | Case Presentation |
| 10c. Important follow-up test results | Case Presentation |
| 10d. Intervention adherence and tolerability | Case Presentation |
| 10e. Adverse and unanticipated events | Case Presentation |
| 11. Discussion – strengths and limitations; relevant literature | Discussion |
| 12. Patient perspective | Not applicable (patient declined) |
| 13. Informed consent – was it obtained? | Consent |
